# Supplementary material for: Supernatant of platelet-Klebsiella pneumoniae coculture induces apoptosis-like death in Klebsiella pneumoniae
Source: Microbiol Spectr. 2024 Jan 30;12(3):e01279-23. doi: 10.1128/spectrum.01279-23 (PMC10913751; doi:10.1128/spectrum.01279-23)
Supplement: Supplemental figures — Fig. S1 to S10. [file spectrum.01279-23-s0001.pdf]

Supplementary Material

Figure S1. Washing and purification of platelets.

(A) The count of platelets after washing and purification. (B) Flow cytometry analysis of CD41a+ cells. (C) Statistical results of CD41a detection in (B). All results have been tested at least three times. Statistical analysis of the data was performed by one-way ANOVA, Tukey multiple comparisons.

Figure S2. Establishment of the optimal system for inhibiting the growth of *K. pneumoniae* with platelet-*K. pneumoniae* coculture supernatant.

(A) Inhibitory effect of platelet-*K. pneumoniae* coculture supernatant prepared with different concentration of platelets on the growth of *K. pneumoniae*. (B) Inhibitory effect of platelet-*K. pneumoniae* coculture supernatant on the growth of different concentration of *K. pneumoniae*. (C) Inhibitory effect of platelet-*K. pneumoniae* coculture supernatant prepared with different concentration of bacteria on the growth of *K. pneumoniae*. All results have been tested at least three times. Statistical analysis of the data was performed by one-way ANOVA, Tukey multiple comparisons.

Figure S3. Antiplatelet agents decreased platelet CD62P expression induced by *K. pneumoniae.*

(A) Platelet CD62P expression was detected by flow cytometry after coculture with *K. pneumoniae*. (B) Statistical results of FITC-CD62P fluorescence intensity in (A). (C) Platelet CD62P expression was detected by flow cytometry after treatment of Aspirin and Ticagrelor in *vitro*. (D) Flow cytometry detection of CD62P expression of platelet pretreated with Aspirin or Ticagrelor for 1 h and then coculture with *K. pneumoniae*. (E) Statistical results of FITC-CD62P fluorescence intensity in (C). (F) Statistical results of FITC-CD62P fluorescence intensity in (D). (G) Analysis of OD_{600nm} in each group. Naked: unstained platelet. Untreated-PLTs： untreated platelets， as a control group. Aspirin treated-PLTs： platelet treated with Aspirin for 1h. Ticagrelor treated

PLTs： platelet treated with Ticagrelor for 1h. Sn+KP: *K. pneumoniae* treated with platelet-*K. pneumoniae* coculture supernatant. Sn(A)+KP: *K. pneumoniae* treated with platelet-*K. pneumoniae* coculture supernatant pretreated with Aspirin. Sn(T)+KP: *K. pneumoniae* treated with platelet-*K. pneumoniae* coculture supernatant pretreated with Ticagrelor. KP: untreated *K. pneumoniae*, as a negative control. IPM: *K. pneumoniae* treated with Imipenem, as a positive control. All results have been tested at least three times. Statistical analysis of the data was performed by one-way ANOVA, Tukey multiple comparisons.

Figure S4. C57BL/6 mice were infected with *K. pneumoniae* by intratracheal injection. (A) Schematic diagram of mice infected with *K. pneumoniae*. (B-E) Detection of the physiological indexes of mice in each group. (B) Changes in body weight of mice. (C) Count analysis of WBCs. (D) Count analysis of PLTs. (E) Detection of Hb level. (F) The number of *K. pneumoniae* in lung and BALF of infected mice in each group. (G) Statistical histogram of bacteria count in lung in (F). (H) Statistical histogram of bacteria count in BALF in (F). (I) Representative images of H&E staining of lung. (J) Statistical histogram of histological score analysis of lung injury in the mice in (I). All results have been tested at least three times. Data presented as mean ± SEM. Statistical analysis of the data was performed by Student's t test.

Figure S5. Detection of physiological indexes of *K. pneumoniae* infected mice.
(A) Changes in body weight of mice. (B) Count analysis of WBCs. (C) Count analysis of PLTs. (D) Detection of Hb level. (E) Statistical histogram of body weight in each group at 48h. (F) Statistical histogram of WBC count in each group at 48 h. (G) Statistical histogram of PLTs count in each group at 48h. (H) Statistical histogram of Hb level in each group at 48h. All results have been tested at least three times. (I) The counts of *K. pneumoniae* in lung and BALF of *K. pneumoniae* infected mice in each group after 48h infection. Statistical analysis of the data was performed by one-way ANOVA, Tukey multiple comparisons.

Figure S6. Changes of biochemical indexes and pH in platelet-*K. pneumoniae* coculture supernatant.

K. pneumoniae were cocultured with or without platelet-*K. pneumoniae* coculture supernatant for 8h. (A) Changes in LDH in each group. (B) Changes in GLU in each group. (C) Changes in the concentration of K^+ in each group. (D) Changes in the concentration of Na^+ in each group. (E) Changes in the concentration of Cl^- in each group. (F) Changes in the concentration of Ca^{2+} in each group. (G) Changes in the concentration of CO_2 in each group. (H) Statistical histogram of LDH level in each group at 8h.(I) Statistical histogram of the concentration of K^+ in each group at 8 h. (J) Statistical histogram of the concentration of Na^+ in each group at 8 h. (K) Statistical histogram of the concentration of Cl^- in each group at 8h. (L) Statistical histogram of the concentration of Ca^{2+} in each group at 8h. (M) Statistical histogram of the concentration of CO_2 in each group at 8h. (N) Statistical histogram of the pH in each group at 8h. All results have been tested at least three times. Statistical analysis of the data was performed by one-way ANOVA, Tukey multiple comparisons.

Figure S7. NAC inhibits the inhibitory effect of platelet-*K. pneumoniae* coculture supernatant on *K. pneumoniae*. (A) The photos of *K. pneumoniae* in each group. (B) Analysis of OD_{600} in each group. (C) The count of *K. pneumoniae* in each group. (D)A schematic diagram of *K. pneumoniae* apoptosis-like death induced by platelet-*K. pneumoniae* coculture supernatant. Sn+KP: *K. pneumoniae* treated with platelet-*K. pneumoniae* coculture supernatant. Sn+KP+NAC: NAC was added to the platelet-*K. pneumoniae* coculture supernatant cocultured with *K. pneumoniae*. NAC+KP: NAC was added to 1640 RPMI medium with *K. pneumoniae*. KP: untreated *K. pneumoniae*, as a negative control. IPM: *K. pneumoniae* treated with Imipenem, as a positive control. Statistical analysis of the data was performed by one-way ANOVA, Tukey multiple comparisons.

Figure S8. Platelet-*K. pneumoniae* coculture supernatant inhibits CRKP growth *in vitro*.

CRKP were cocultured with or without platelet-*K. pneumoniae* coculture supernatant for 8h. (A) The photos of CRKP in each group. (B) Analysis of OD_{600nm} in each group. (C) The count of CRKP in each group. CRKP: untreated, as a negative control. Sn+CRKP: CRKP cocultured with platelet-*K. pneumoniae* coculture supernatant. IPM: CRKP treated with Imipenem, as a negative control. TGC: CRKP treated with Tigecycline, as a positive control. All results have been tested at least three times. Statistical analysis of the data was performed by one-way ANOVA, Tukey multiple comparisons.

Figure S9. Identification of resistance and virulence genes to CRKP.

CRKP (clinical): Clinically isolated CRKP strains. (A) Identification of CRKP phenotypic resistance with IPM and TGC. (B) The results of agarose gel nucleic acid electrophoresis identification after amplification of *bla*$_{KPC}$ gene of CRKP. (C) Identification of virulence genes of CRKP. (D) The photo of MIC of TGC and IPM for CRKP clinically isolated strains using well plate. (E) The OD_{600nm} of MIC of TGC for CRKP clinical isolated strains. (F) The OD_{600nm} of MIC of IPM for CRKP clinical isolated strains. All results have been tested at least three times. Statistical analysis of the data was performed by one-way ANOVA, Tukey multiple comparisons.

Figure S10. Detection of physiological indexes of CRKP infected mice.

(A) Changes in body weight of mice. (B) Count analysis of WBCs. (C) Count analysis of PLTs. (D) Detection of Hb level. (E) Statistical histogram of body weight in each group at 48h. (F) Statistical histogram of WBCs count in each group at 48h. (G) Statistical histogram of PLTs count in each group at 48h. (H) Lung of mice in each group. (I) The number of CRKP in lung and BALF of infected mice in each group after 48h infection. All results have been tested at least three times. Statistical analysis of the data was performed by one-way ANOVA, Tukey multiple comparisons.

FIG S1

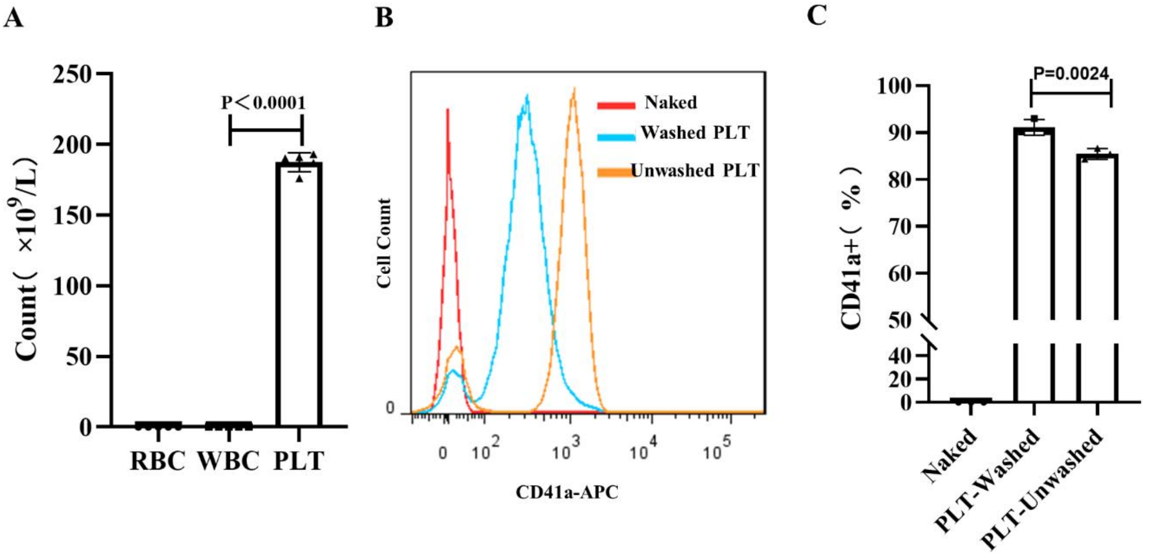
A
B
C
Count（ ×10⁹/L)
250
200
150
100
50
0
P＜0.0001
RBC
WBC
PLT
Naked
Washed PLT
Unwashed PLT
Cell Count
CD41a-APC
CD41a+（ %）
P=0.0024
Naked
PLT-Washed
PLT-Unwashed

FIG S2

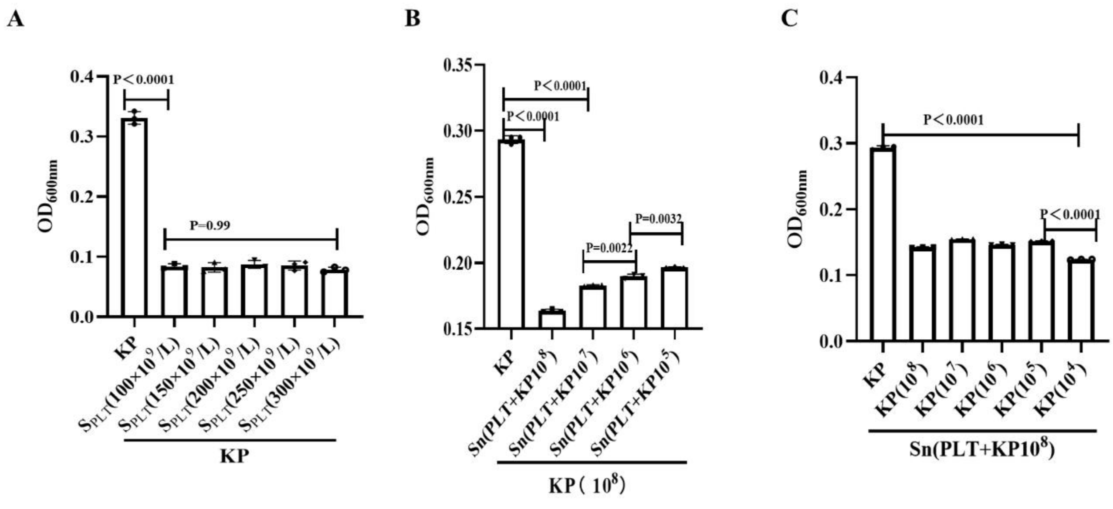
A
B
C
OD600nm
P＜0.0001
P=0.99
KP
P=0.0022
P=0.0032
KP（10⁸）
Sn(PLT+KP10⁸)

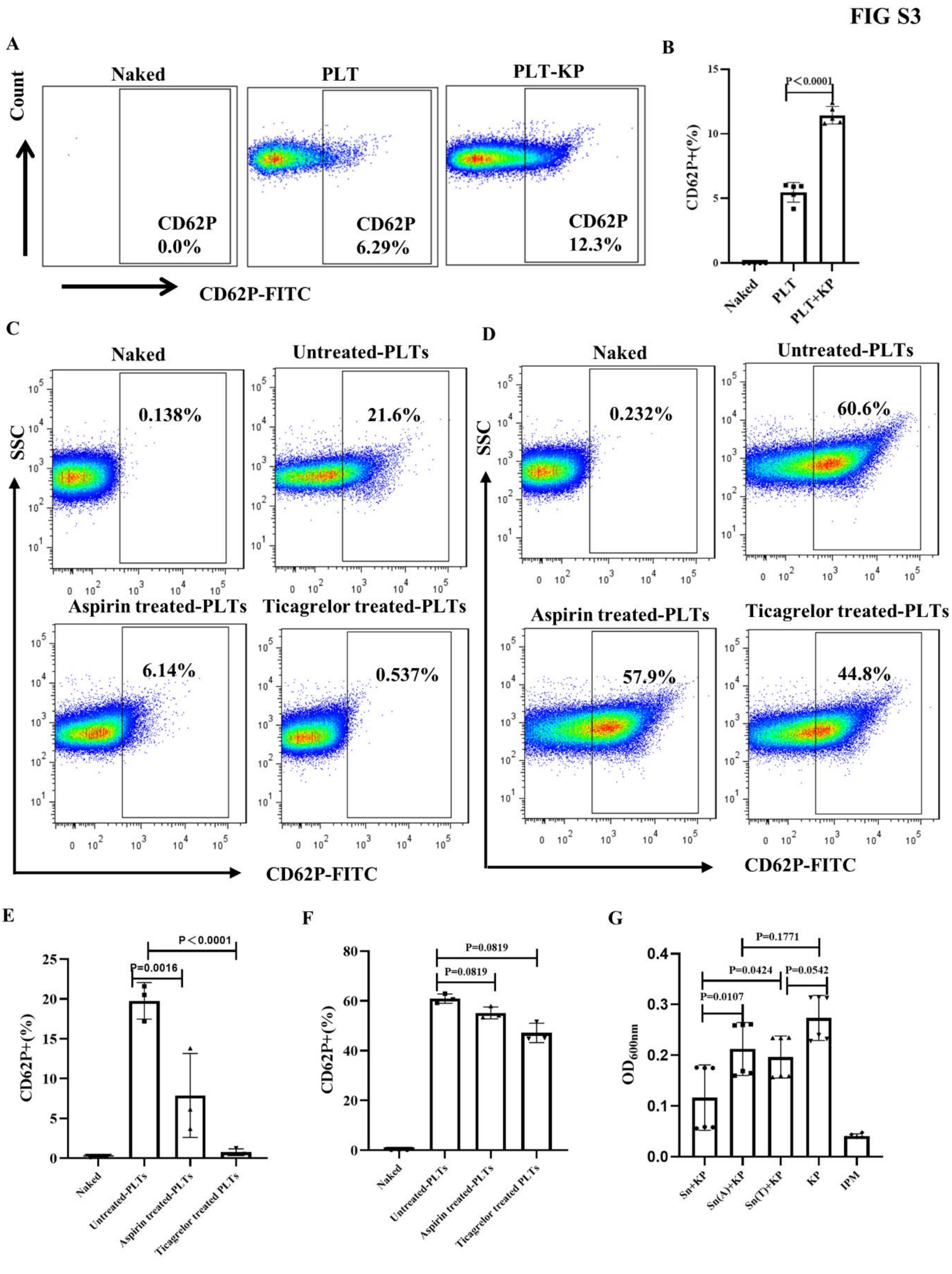

FIG S3
A
Naked
PLT
PLT-KP
Count
CD62P 0.0%
CD62P 6.29%
CD62P 12.3%
CD62P-FITC
B
CD62P+(%)
P＜0.0001
Naked
PLT
PLT+KP
C
Naked
Untreated-PLTs
0.138%
21.6%
Aspirin treated-PLTs
Ticagrelor treated-PLTs
6.14%
0.537%
SSC
CD62P-FITC
D
Naked
Untreated-PLTs
0.232%
60.6%
Aspirin treated-PLTs
Ticagrelor treated-PLTs
57.9%
44.8%
SSC
CD62P-FITC
E
CD62P+(%)
P=0.0016
P＜0.0001
Naked
Untreated-PLTs
Aspirin treated-PLTs
Ticagrelor treated PLTs
F
CD62P+(%)
P=0.0819
P=0.0819
Naked
Untreated-PLTs
Aspirin treated-PLTs
Ticagrelor treated PLTs
G
OD600nm
P=0.1771
P=0.0424
P=0.0542
P=0.0107
Sn+KP
Sn(A)+KP
Sn(T)+KP
KP
IPM

FIG S4

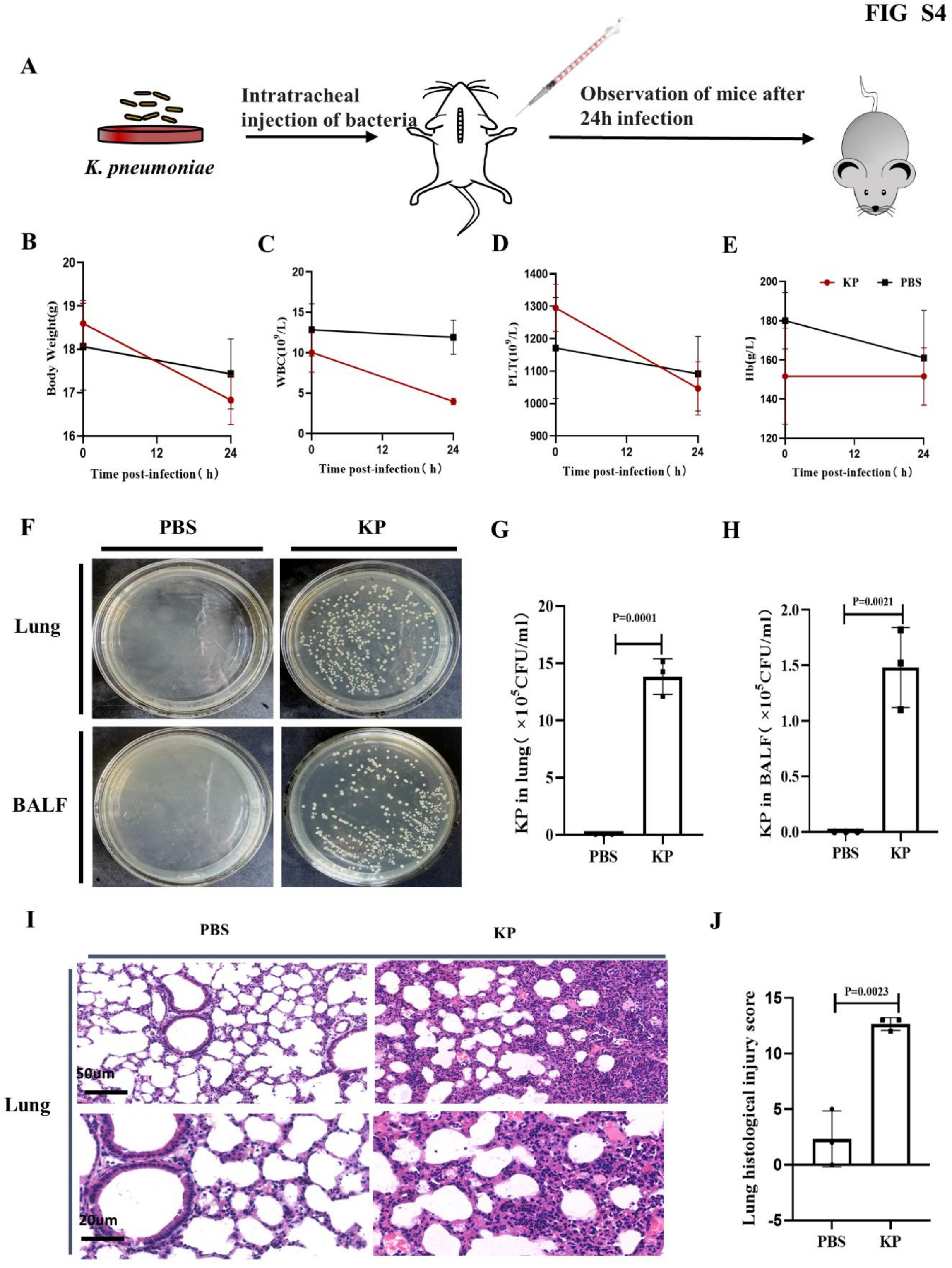
A
K. pneumoniae
Intratracheal injection of bacteria
Observation of mice after 24h infection
B
Body Weight(g)
Time post-infection (h)
C
WBC(10^9/L)
Time post-infection (h)
D
PLT(10^9/L)
Time post-infection (h)
E
Hb(g/L)
KP
PBS
Time post-infection (h)
F
PBS
KP
Lung
BALF
G
P=0.0001
KP in lung (×10^5CFU/ml)
PBS
KP
H
P=0.0021
KP in BALF (×10^5CFU/ml)
PBS
KP
I
PBS
KP
Lung
50um
20um
J
P=0.0023
Lung histological injury score
PBS
KP

FIG S5

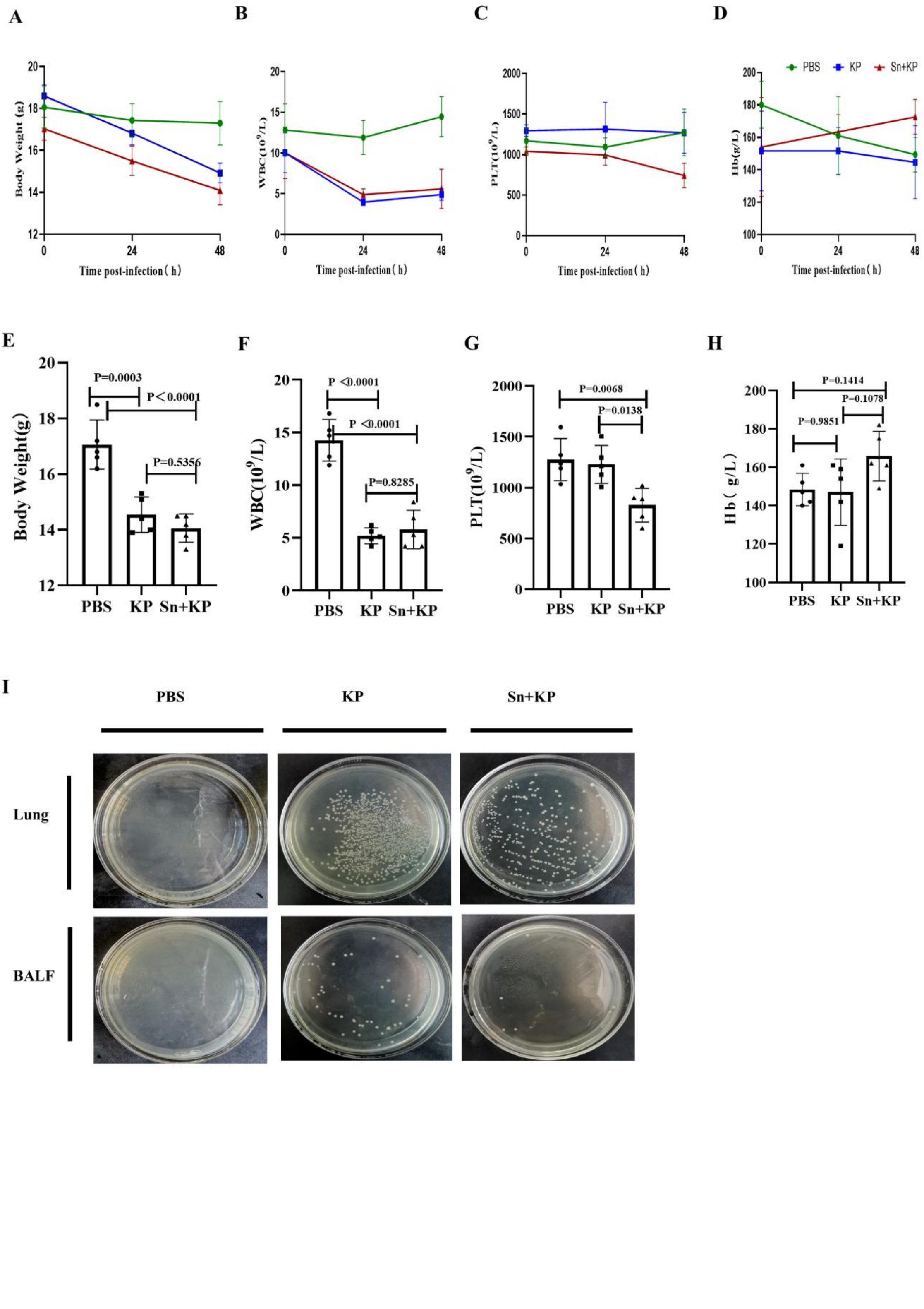
A
B
C
D
PBS
KP
Sn+KP
Body Weight (g)
WBC(10⁹/L)
PLT(10⁹/L)
Hb(g/L)
Time post-infection (h)
E
F
G
H
P=0.0003
P＜0.0001
P=0.5356
P <0.0001
P <0.0001
P=0.8285
P=0.0068
P=0.0138
P=0.1414
P=0.1078
P=0.9851
Body Weight(g)
WBC(10⁹/L)
PLT(10⁹/L)
Hb (g/L)
I
Lung
BALF

FIG S6

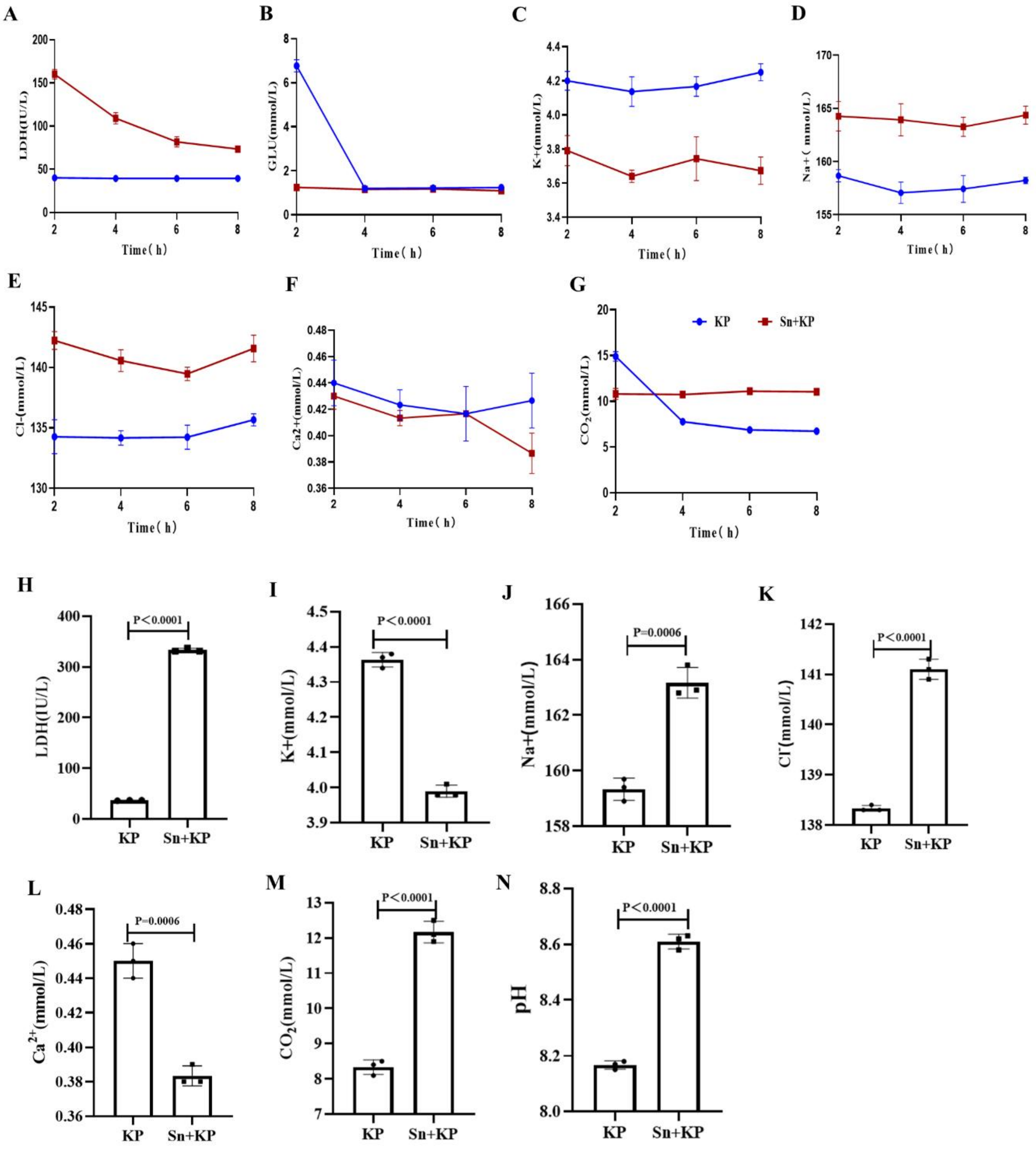
A
B
C
D
LDH(IU/L)
GLU(mmol/L)
K+(mmol/L)
Na+(mmol/L)
Time(h)
E
F
G
Cl-(mmol/L)
Ca2+(mmol/L)
CO2(mmol/L)
KP
Sn+KP
Time(h)
H
I
J
K
P<0.0001
P=0.0006
LDH(IU/L)
K+(mmol/L)
Na+(mmol/L)
Cl-(mmol/L)
KP
Sn+KP
L
M
N
P=0.0006
P<0.0001
Ca2+(mmol/L)
CO2(mmol/L)
pH

FIG S7

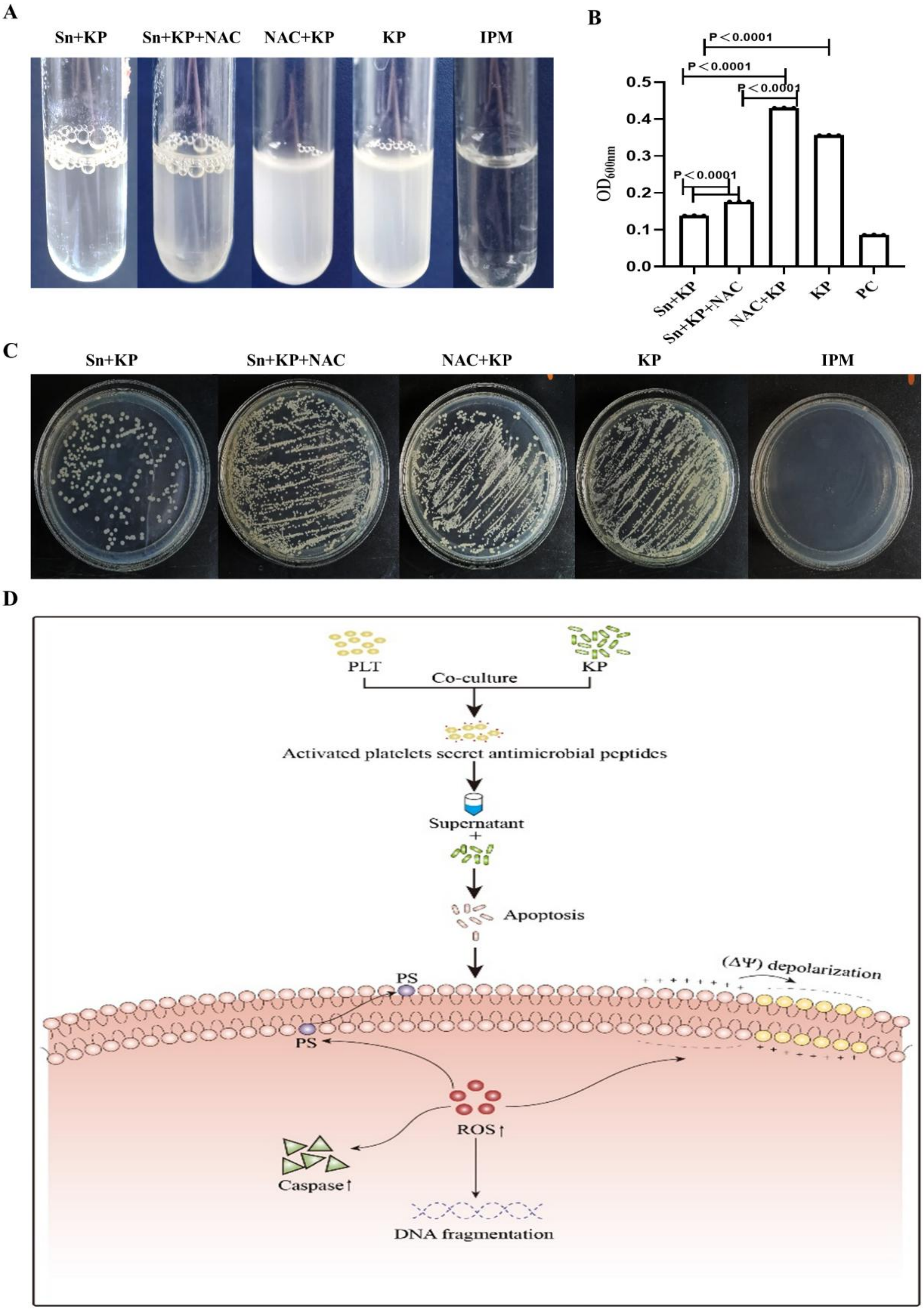
A
Sn+KP
Sn+KP+NAC
NAC+KP
KP
IPM
B
P＜0.0001
P＜0.0001
P＜0.0001
P＜0.0001
OD600nm
0.5
0.4
0.3
0.2
0.1
0.0
Sn+KP
Sn+KP+NAC
NAC+KP
KP
PC
C
Sn+KP
Sn+KP+NAC
NAC+KP
KP
IPM
D
PLT
Co-culture
KP
Activated platelets secret antimicrobial peptides
Supernatant
+
Apoptosis
PS
(ΔΨ) depolarization
PS
ROS↑
Caspase↑
DNA fragmentation

FIG S8

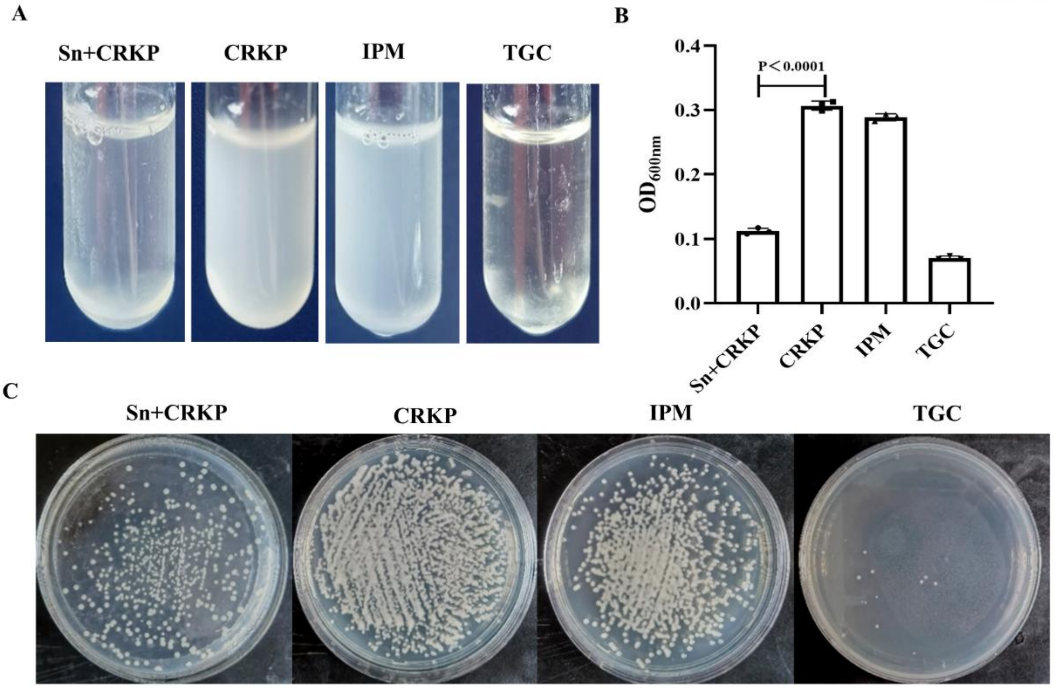
A
Sn+CRKP
CRKP
IPM
TGC
B
P < 0.0001
OD600nm
0.4
0.3
0.2
0.1
0.0
Sn+CRKP
CRKP
IPM
TGC
C
Sn+CRKP
CRKP
IPM
TGC

FIG S9

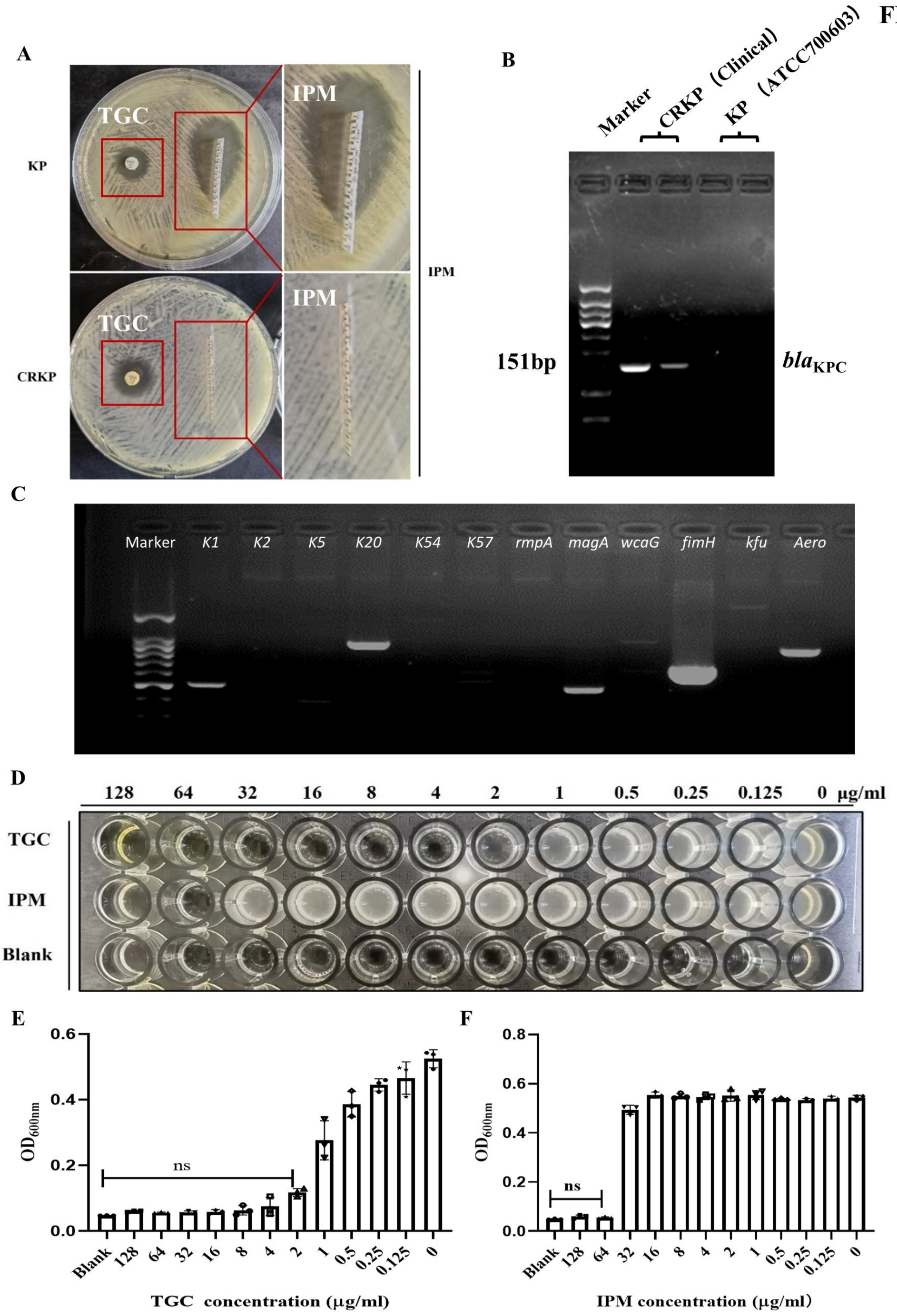

A
TGC
IPM
KP
CRKP
IPM
B
Marker
CRKP（Clinical）
KP（ATCC700603）
151bp
blaKPC
C
Marker
K1
K2
K5
K20
K54
K57
rmpA
magA
wcaG
fimH
kfu
Aero
D
128
64
32
16
8
4
2
1
0.5
0.25
0.125
0 μg/ml
TGC
IPM
Blank
E
OD600nm
ns
TGC concentration (μg/ml)
F
OD600nm
ns
IPM concentration (μg/ml)

FIG S10

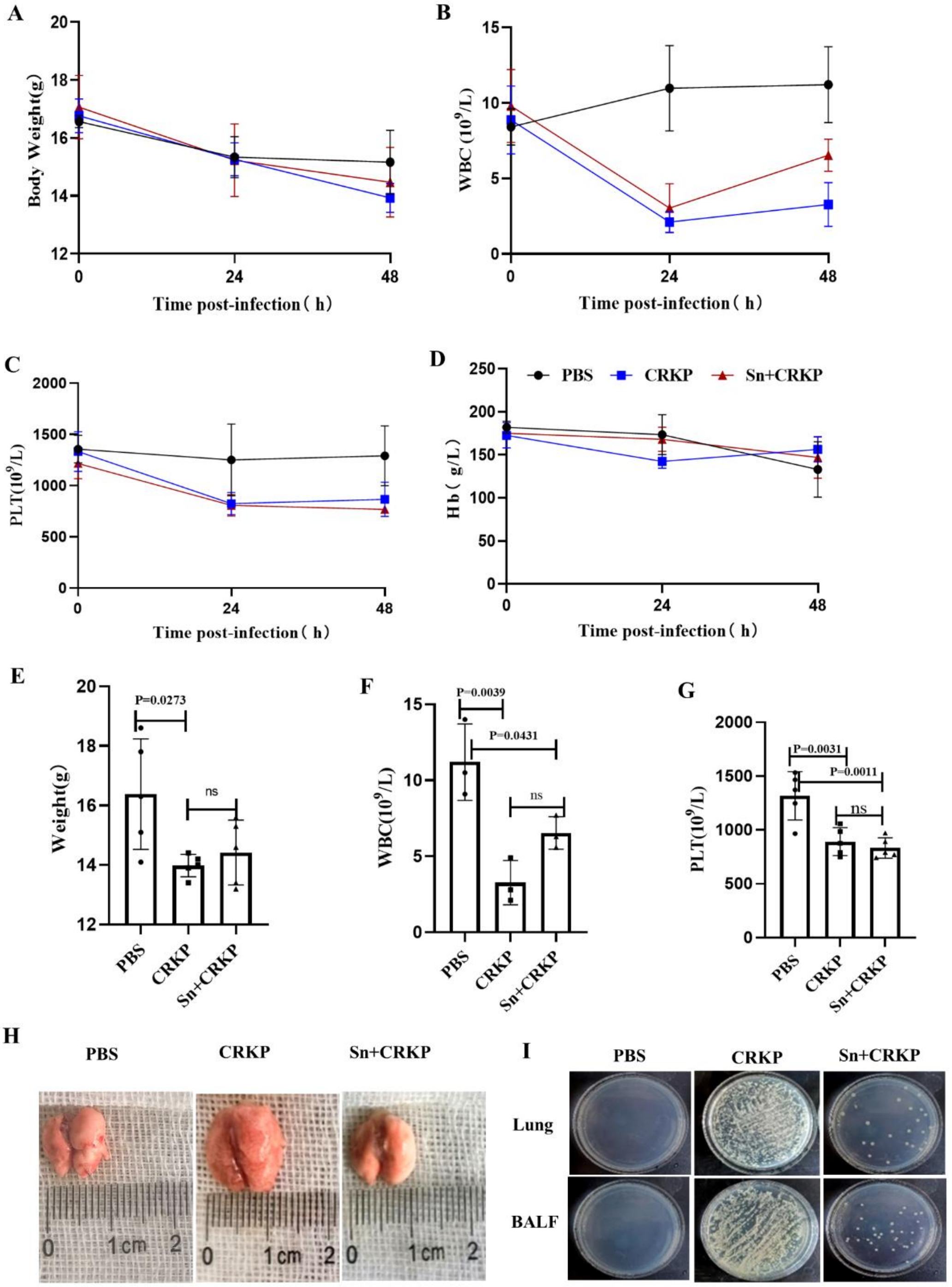

A
Body Weight(g)
Time post-infection（h）
B
WBC (10^9/L)
Time post-infection（h）
C
PLT(10^9/L)
Time post-infection（h）
D
PBS
CRKP
Sn+CRKP
Hb（g/L）
Time post-infection（h）
E
Weight(g)
P=0.0273
ns
PBS
CRKP
Sn+CRKP
F
WBC(10^9/L)
P=0.0039
P=0.0431
ns
G
PLT(10^9/L)
P=0.0031
P=0.0011
ns
H
PBS
CRKP
Sn+CRKP
I
PBS
CRKP
Sn+CRKP
Lung
BALF
